# Predictive value of Th17/Treg immune imbalance for disease severity and poor prognosis in children with respiratory syncytial virus pneumonia

**DOI:** 10.3389/fped.2025.1725402

**Published:** 2025-12-04

**Authors:** Minjuan Zhu, Jian Liu

**Affiliations:** Department of Pediatrics, The People’s Hospital of Suzhou New District, Suzhou, Jiangsu, China

**Keywords:** Th17/Treg ratio, immune imbalance, respiratory syncytial virus pneumonia, disease severity, prognosis, logistic regression, receiver operating characteristic curve

## Abstract

**Objective:**

Respiratory syncytial virus pneumonia (RSVP) remains a major cause of pediatric hospitalization. This study evaluates the value of Th17/Treg imbalance in assessing disease severity and predicting prognosis among RSVP children.

**Methods:**

RSVP children (422 cases) during May 2022–May 2024 were retrospectively enrolled and divided into mild and moderate-to-severe groups based on disease severity. Additionally, 358 healthy children were recruited (the control group). Peripheral blood Th17 and Treg cell proportions were quantified by flow cytometry, with the Th17/Treg ratio calculated. Serum IL-17, IL-6, IL-10, and TGF-β1 levels were measured using ELISA. Logistic regression models were established to identify risk factors for poor prognosis. ROC curves were plotted to evaluate predictive performance.

**Results:**

RSVP children demonstrated higher Th17 cell proportion, IL-17 and IL-6 levels, and Th17/Treg ratio than controls, alongside lower Treg cell proportion and IL-10 and TGF-β1 levels (all *P* < 0.001). These alterations correlated with disease severity and prognosis. Th17/Treg ratio and IL-17 were independent risk factors for poor prognosis, while IL-10 was a protective factor (all *P* < 0.05). The Th17/Treg ratio yielded an AUC of 0.831 (77.05% sensitivity, 77.01% specificity) for predicting poor prognosis. AUCs were 0.770 for IL-17 (67.21% sensitivity, 78.67% specificity) and 0.756 for IL-10 (57.38% sensitivity, 83.38% specificity). The combined model (Th17/Treg ratio + IL-17 + IL-10) achieved a superior AUC of 0.919 (93.44% sensitivity, 75.35% specificity; all *P* < 0.001).

**Conclusion:**

Peripheral blood Th17/Treg imbalance is a characteristic of RSVP children. The combined detection of Th17/Treg ratio, IL-17, and IL-10 can help predict poor prognosis in affected children.

## 1 Introduction

Respiratory syncytial virus (RSV) stands as the leading global pathogen responsible for acute lower respiratory tract infections (ALRTIs) in children under five years of age, presenting a major public health challenge (1). RSV infection manifests across a broad clinical spectrum, ranging from mild upper respiratory symptoms to severe pneumonia. Critically ill cases may progress to respiratory failure, heart failure, and even death, with disproportionately high morbidity and mortality rates observed in infants and young children (2). Despite ongoing advancements in the clinical diagnosis and management of RSV pneumonia (RSVP), including optimized antiviral therapies and supportive care (3), the persistent lack of reliable biological markers for accurately assessing disease severity and predicting outcomes significantly hinders the development of personalized treatment strategies and the improvement of clinical endpoints.

Recent immunological investigations have revealed that immune dysregulation plays a central role in the pathogenesis of RSVP (4). Specifically, the dynamic equilibrium among CD4^+^ T cell subsets, such as T helper (Th) 1, Th2, Th17, and regulatory T (Treg) cells, is crucial for regulating the post-infection immune response (5). Th17 cells, a key pro-inflammatory subset, contribute to host's defense against extracellular pathogens through the secretion of cytokines including interleukin (IL)-17 and IL-6. However, their excessive activation can trigger neutrophil infiltration and cytokine storms, exacerbating lung tissue damage (6). Conversely, Treg cells maintain immune homeostasis and mitigate damage from excessive immune responses by producing inhibitory cytokines such as IL-10 and transforming growth factor-β (TGF-β) (7). As an important anti-inflammatory cytokine, IL-10 can also promote the stabilization, expansion, and functional maintenance of Treg cells through its signaling pathways (e.g., via mechanisms such as STAT5 phosphorylation) (8). Under physiological conditions, a delicate balance between Th17 and Treg cells, mediated by cytokine networks and cross-regulation of transcription factors, ensures the precision of immune responses (9). Multiple studies have confirmed that RSV infection disrupts this critical balance. Clinical research demonstrates that children with RSVP exhibit significantly elevated proportions of Th17 cells and reduced proportions of Treg cells in peripheral blood and airway secretions. Moreover, the Th17/Treg ratio positively correlates with disease severity (10). Animal models further validate these findings, showing increased Th17 cell infiltration and impaired Treg cell function in the lungs of RSV-infected mice, leading to aggravated alveolitis and airway hyperresponsiveness (11). These findings suggest that RSV induces airway epithelial cells to secrete chemokines, which promote Th17 cell differentiation while suppressing Treg cell function, establishing a vicious cycle of pro-inflammatory and anti-inflammatory imbalance.

Although an association between Th17/Treg imbalance and RSVP severity has been established, a critical gap remains in translating this immunological finding into clinically actionable tools. Currently, there is a lack of validated models for individual prognosis prediction using Th17/Treg-related parameters. Therefore, the primary novelty and objective of this study extend beyond merely reiterating this association. Our objective is to bridge this translational gap by systematically evaluating the prognostic predictive value of Th17/Treg immune parameters.

## 2 Materials and methods

### 2.1 Study subjects

A retrospective cohort of 487 pediatric patients with RSVP admitted to The People's Hospital of Suzhou New District between May 2022 and May 2024 was initially selected. Following the inclusion and exclusion criteria, 422 children were ultimately enrolled as study subjects. These participants were divided into mild and moderate-to-severe groups according to disease severity criteria outlined in the Guidelines for the Management of Community-Acquired Pneumonia in Children (12, 13). Additionally, 358 children undergoing routine health examinations at The People's Hospital of Suzhou New District were recruited as the control group. The study protocol received approval from the Ethics Committee of The People's Hospital of Suzhou New District and conformed to the principles of the *Declaration of Helsinki*.

### 2.2 Inclusion and exclusion criteria

Inclusion criteria comprised: (1) virologically confirmed RSVP via positive results for the RSV immunofluorescence assay; (2) age between 1 and 24 months; (3) disease course <48 h; and (4) complete medical records.

Exclusion criteria encompassed: (1) concurrent other viral respiratory infections or secondary bacterial infections (Microbiological, inflammatory marker, or imaging evidence of bacterial pneumonia following a confirmed RSV diagnosis). ① Pathogenic bacteria isolated from sputum culture, blood culture, or bronchoalveolar lavage fluid. ② Chest x-ray or CT showing new or progressive consolidation, cavitation, pleural effusion, or other findings suggestive of bacterial pneumonia. ③ Serum procalcitonin (PCT) ≥0.5 ng/ml or significantly elevated C-reactive protein (CRP) (e.g., >50 mg/L) without other explanations. ④ Clinical judgment: Comprehensive assessment by two or more attending physicians confirming secondary bacterial infection, with antimicrobial therapy already initiated; (2) children with obesity or malnutrition; (3) pneumonia induced by other etiologies; (4) bronchial or pulmonary dysplasia; (5) comorbid immunosuppressive conditions; and (6) administration of corticosteroids or other medications affecting study outcomes within the preceding 3 months.

### 2.3 Disease severity assessment

RSVP children were stratified into mild and moderate-to-severe groups according to the Guidelines for the Management of Community-Acquired Pneumonia in Children (12, 13). The mild group encompassed children presenting with upper respiratory tract infection symptoms such as nasal congestion, rhinorrhea, low-grade fever, and cough, who were deemed suitable for outpatient or home management. Conversely, the moderate-to-severe group included children exhibiting marked dyspnea, wheezing/distressed breathing, circumoral cyanosis, nasal flaring, and three-concave sign, all requiring hospitalization for comprehensive medical intervention.

### 2.4 Clinical data collection

Data including age, sex, virus subtype, fever severity, birth weight, body weight, mode of delivery, and gestational age were retrieved and collated through systematic review of electronic medical records. For all participants, 10 ml of fasting antecubital venous blood was obtained on the day of hospital admission in the absence of any prior therapeutic intervention. A portion of the peripheral blood sample underwent immediate flow cytometric analysis, while the remainder was processed to isolate serum, which was cryopreserved for subsequent biochemical analyses.

### 2.5 Quantification of peripheral blood Th17 and Treg cells

Peripheral blood Th17 and Treg cell frequencies were determined by flow cytometry (CytoFLEX, Beckman Coulter, Brea, CA, USA) as previously described (14), with Th17 cells defined as CD4^+^CD196^+^ and Treg cells as CD4^+^CD25^+^FOXP3^+^. Briefly, cells suspended in 1 ml phosphate-buffered saline (PBS) were stimulated with 2 μl cell activation cocktail at 37°C for 4 h. Following centrifugation at 350 × g for 5 min, pellets were resuspended in 100 μl PBS and labeled with CD4 (5 μl/tube) and CD25 (5 μl/tube) in the dark at 4°C for 30 min, with the supernatant discarded. Cells were then washed with PBS and centrifuged (350 × g, 5 min) to remove the supernatant. Fixation was performed using 1 ml mixture of True-Nuclear™ 4X fix concentrate and True-Nuclear™ fix diluent at 1:3 during a 50-minute incubation at room temperature in darkness. After centrifugation (400 × g, 5 min) for supernatant removal, cells were resuspended with 1 ml/tube of 1X permeabilization-wash buffer and centrifuged at 400 × g for 5 min, which was repeated twice. Cell pellets were subsequently resuspended in 100 μl Perm buffer working solution and labeled with CD196 (5 μl) and FOXP3 (5 μl) at 4°C for 30 min protected from light. Following two washes with 1 ml PBS and centrifugation (400 × g, 5 min), cells were resuspended in 500 μl PBS for flow cytometric acquisition to quantify CD4^+^CD196^+^ and CD4^+^CD25^+^FOXP3^+^ populations.

The following reagents from BioLegend (San Diego, CA, USA) were employed: FITC anti-human CD4 antibody (catalog #300505), APC anti-human CD196 (CCR6) antibody (#353416), APC anti-human CD25 antibody (#302609), PE anti-mouse/rat/human FOXP3 antibody (#320007), True-Nuclear™ transcription factor buffer set (#424401), permeabilization wash buffer (#421002), and cell activation cocktail (#423303).

### 2.6 Enzyme-linked immunosorbent assay (ELISA)

Serum levels of Th17-associated cytokines IL-17 (catalog #ml058051) and IL-6 (#ml058097), as well as Treg-associated anti-inflammatory cytokines IL-10 (#ml064299) and TGF-β1 (#ml022522-2) were quantified using ELISA kits (MLBio, Shanghai, China) on a Multiskan Mk3 automated microplate reader (Thermo Scientific, MA, USA). These kits demonstrated high sensitivity and specificity with no cross-reactivity to other cytokines, exhibiting both intra- and inter-assay coefficients of variation below 10%.

### 2.7 Prognostic evaluation

All enrolled children received symptomatic treatment including oxygen supply, antipyretics, bronchodilators, antitussives, and antiviral therapy. Treatment efficacy was assessed after 7 days according to established criteria (12). Prognosis assessment is a comprehensive clinical judgment primarily based on improvements in the following four aspects: ① Clinical symptoms; ② Laboratory inflammatory markers; ③ Imaging studies; ④ Control of complications. The details are as follows:

Cure: complete resolution of clinical symptoms, normalization of laboratory parameters (including but not limited to complete blood count, CRP, and PCT), full radiographic resolution of infiltrates, and absence or complete resolution of complications.
Marked improvement: meeting ≥3 of the following criteria: partial symptomatic relief including reduced cough severity and normalized respiratory rate, significant improvement in laboratory parameters (For example: CRP decrease ≥50%, PCT <0.1 ng/ml, and/or white blood cell count returning to normal range), ≥50% radiographic resolution, or stabilized complications such as improved oxygenation indices without achieving ventilator liberation in respiratory failure cases.
Improvement: meeting ≥2 criteria: mild symptomatic relief (decreased but unresolved fever), partial recovery of laboratory parameters (For example, white blood cell count is approaching normal levels, or inflammatory markers have improved compared to previous readings but have not yet met the criteria for significant improvement), absence of radiographic progression, or non-worsening complications (no new organ dysfunction).
Ineffective: Meeting any of the following criteria: clinical symptoms show no improvement or worsening (e.g., respiratory failure progressing to ARDS), laboratory indicators deteriorate (e.g., persistent elevation of CRP or emergence of new laboratory abnormalities), imaging studies reveal enlargement of existing lesions or new infiltrates, or complications progress (e.g., myocarditis developing into heart failure).

Patients who achieved cure, marked improvement, and improvement were assigned to the favorable prognosis group, while those with ineffective response were assigned to the unfavorable prognosis group.

### 2.8 Statistical analysis

Sample size estimation was performed using G*Power software version 3.1.9.7 (Heinrich Heine University, Düsseldorf, Germany), confirming that the enrolled cohort met the requirements for independent sample *t*-tests, Mann–Whitney *U*-tests, and chi-square (χ^2^) tests. All statistical computations and graphical representations were executed with SPSS Statistics version 27.0 (IBM Corp., Armonk, NY, USA) and GraphPad Prism version 9.5 (GraphPad Software Inc., San Diego, CA, USA) software. Normality of variables was assessed via the Kolmogorov–Smirnov test. Normally distributed measurement data were presented as mean ± standard deviation and compared between groups using independent sample *t*-tests. Non-normally distributed measurement data were expressed as median (minimum, maximum) and analyzed with Mann–Whitney *U*-tests. Enumeration data were summarized as number and percentage, with intergroup comparisons performed using χ^2^ tests. The receiver operating characteristic (ROC) curve analysis was employed to evaluate the predictive value of Th17/Treg imbalance for poor prognosis in RSVP children. Comparisons of multiple area under the curve (AUC) values were conducted using the DeLong's test within MedCalc software version 20.0.15 (MedCalc Software Ltd., Ostend, Belgium). Logistic regression modeling was subsequently implemented to identify independent risk factors for unfavorable prognosis in the RSVP cohort. All reported *P*-values corresponded to two-tailed tests, with statistical significance defined as *P* < 0.05.

## 3 Results

### 3.1 Comparisons of baseline characteristics

No statistically significant difference was observed between the RSVP and control groups regarding sex, age, body weight, mode of delivery, gestational age, or birth weight (all *P* > 0.05), as detailed in Table 1.

**Table 1:** Comparisons of baseline characteristics.

Indicator	Control group (*n* = 358)	RSVP group (*n* = 422)	*t*/Z/χ^2^	*P*
Sex (male/female, *n*)	196/162	234/188	0.039	0.844
Age (months)	7 (2, 24)	7 (2, 22)	0.800	0.424
Body weight (kg)	8.14 (2.98, 15.05)	8.00 (3.04, 13.79)	1.233	0.218
Mode of delivery (vaginal delivery/caesarean section, *n*)	219/139	255/167	0.045	0.831
Gestational age (weeks)	39 (36, 42)	39 (36, 42)	1.720	0.085
Birth weight (kg)	3.19 ± 0.35	3.23 ± 0.29	1.719	0.086
Virus subtype (A/B, *n*)	–	224/198	–	–
Fever severity (moderate/high, *n*)	–	239/183	–	–

The normality was assessed using the Kolmogorov–Smirnov test. Normally distributed measurement data were presented as mean ± standard deviation and compared using the independent sample *t*-test. Non-normally distributed measurement data were presented as median (minimum, maximum) and compared using the Mann–Whitney *U*-test. Enumeration data were presented as number and percentage and compared using the χ^2^ test.

### 3.2 Peripheral blood Th17/Treg immune imbalance in RSVP children

**Figure 1:**
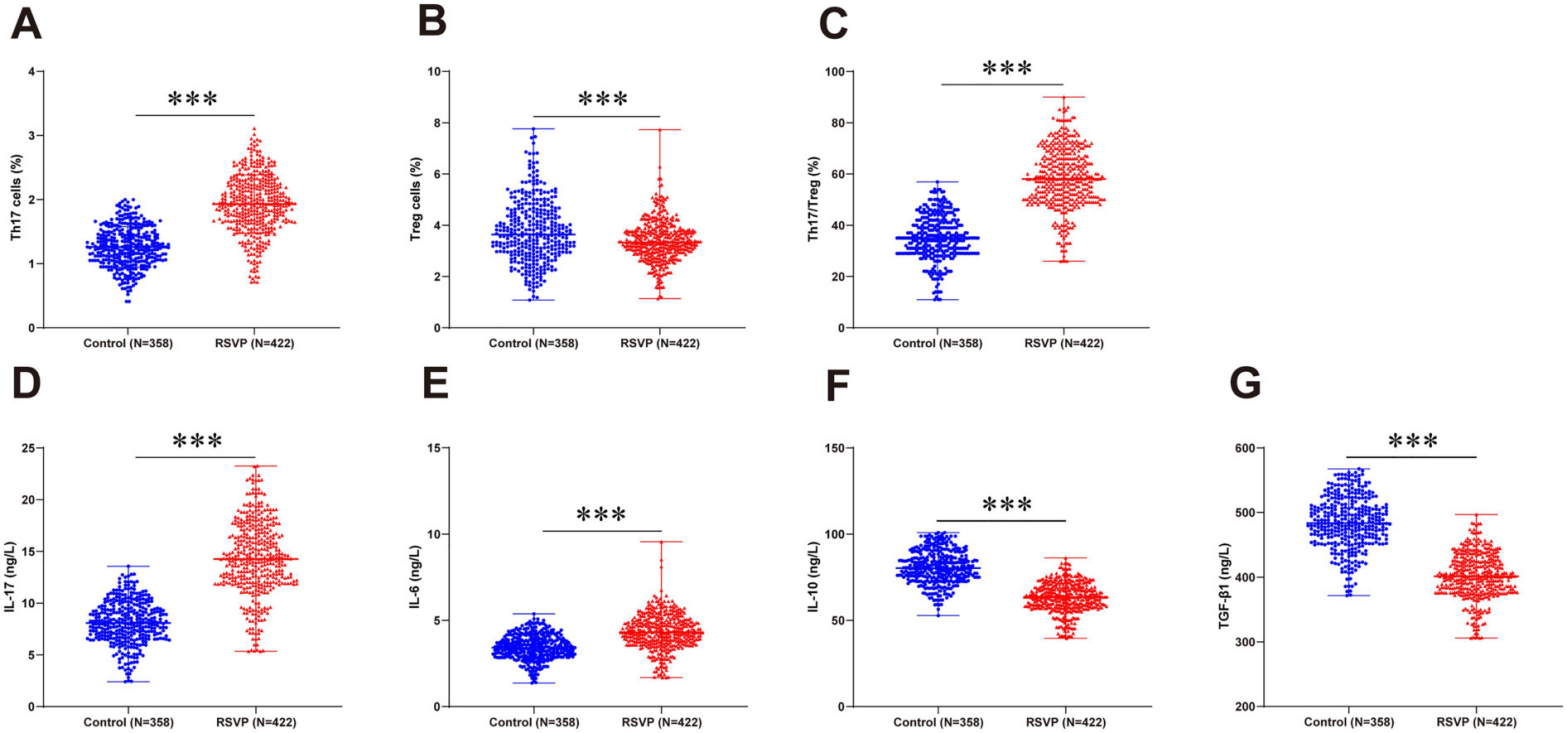
Peripheral blood Th17/Treg immune imbalance in RSVP children. Peripheral blood Th17 **(A)** and Treg **(B)** cell proportions determined by flow cytometry, with the Th17/Treg ratio **(C)** calculated. Serum levels of IL-17 **(D)**, IL-6 **(E)**, IL-10 **(F)**, and TGF-β1 **(G)** measured by ELISA. The normality was assessed using the Kolmogorov–Smirnov test. Data in the panel **(A)** (normally distributed) were presented as mean ± standard deviation and compared by the independent sample *t*-test. Data in panels **(B–G)** (non-normally distributed) were expressed as median (minimum, maximum) and analyzed by the Mann–Whitney *U*-test. *** *P* < 0.001.

The flow cytometric analysis of peripheral blood revealed a significantly elevated proportion of Th17 cells and a reduced proportion of Treg cells in RSVP patients compared to controls. Consequently, the Th17/Treg ratio was markedly increased in the RSVP group. Concordant with these cellular findings, ELISA demonstrated substantially higher serum concentrations of Th17-associated cytokines (IL-17 and IL-6) but lower levels of Treg-associated anti-inflammatory cytokines (IL-10 and TGF-β1) in RSVP patients relative to healthy children (all *P* < 0.001, Figure 1).

### 3.3 Association of Th17/Treg balance with viral subtypes, disease severity, and prognosis in RSVP children

**Figure 2:**
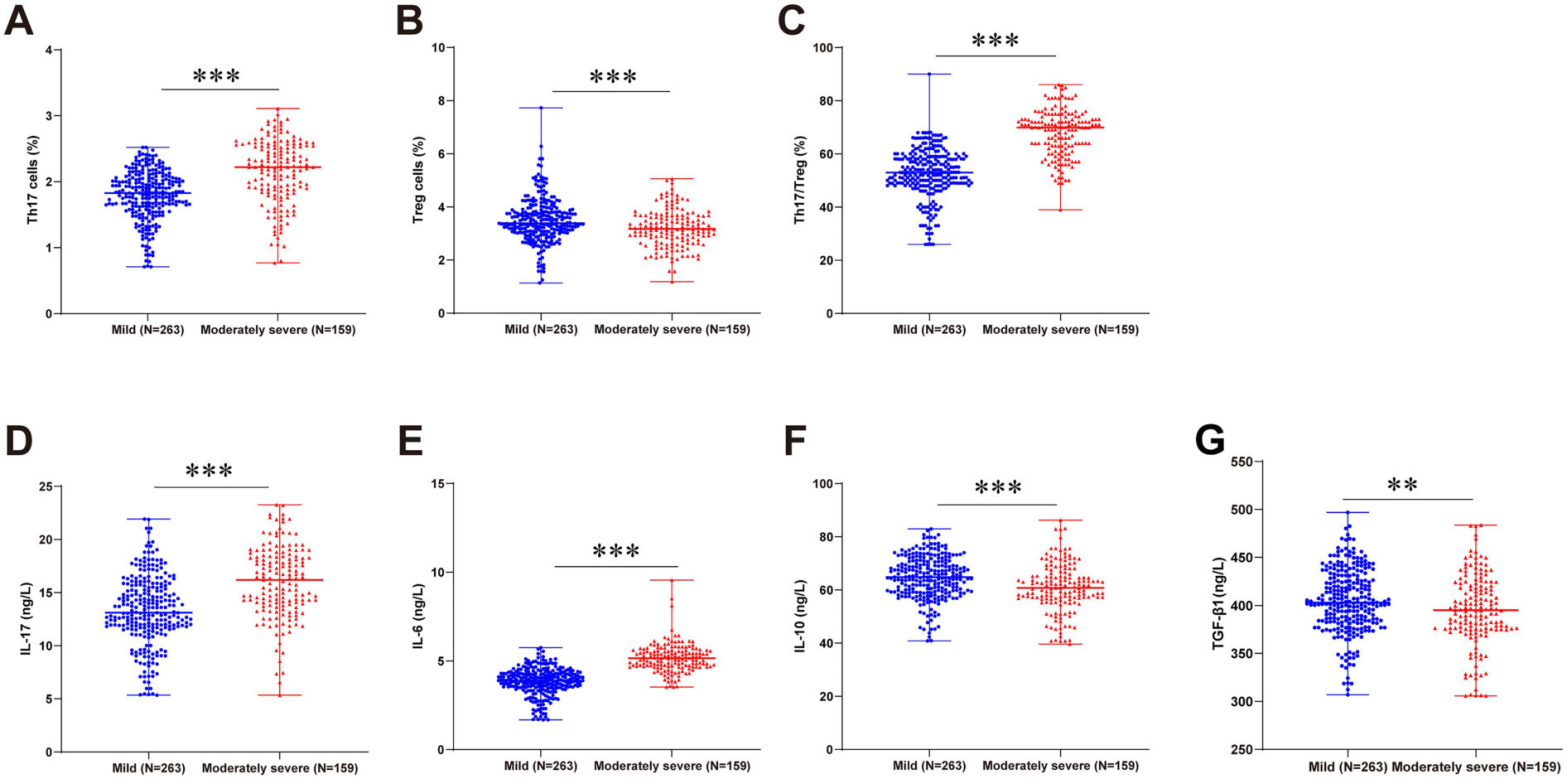
Th17/Treg balance and cytokine profiles stratified by RSVP severity. RSVP children were categorized into mild and moderate-to-severe groups based on disease severity. Flow cytometric quantification of Th17 **(A)** and Treg **(B)** cell proportions, and calculation of the Th17/Treg ratio **(C)** ELISA-measured serum levels of IL-17 **(D)**, IL-6 **(E)**, IL-10 **(F)**, and TGF-β1 **(G)** For panels **(A–G)**, non-normally distributed measurement data were expressed as median (minimum, maximum) and compared using the Mann–Whitney *U*-test. * *P* < 0.05, ** *P* < 0.01, *** *P* < 0.001.

**Figure 3:**
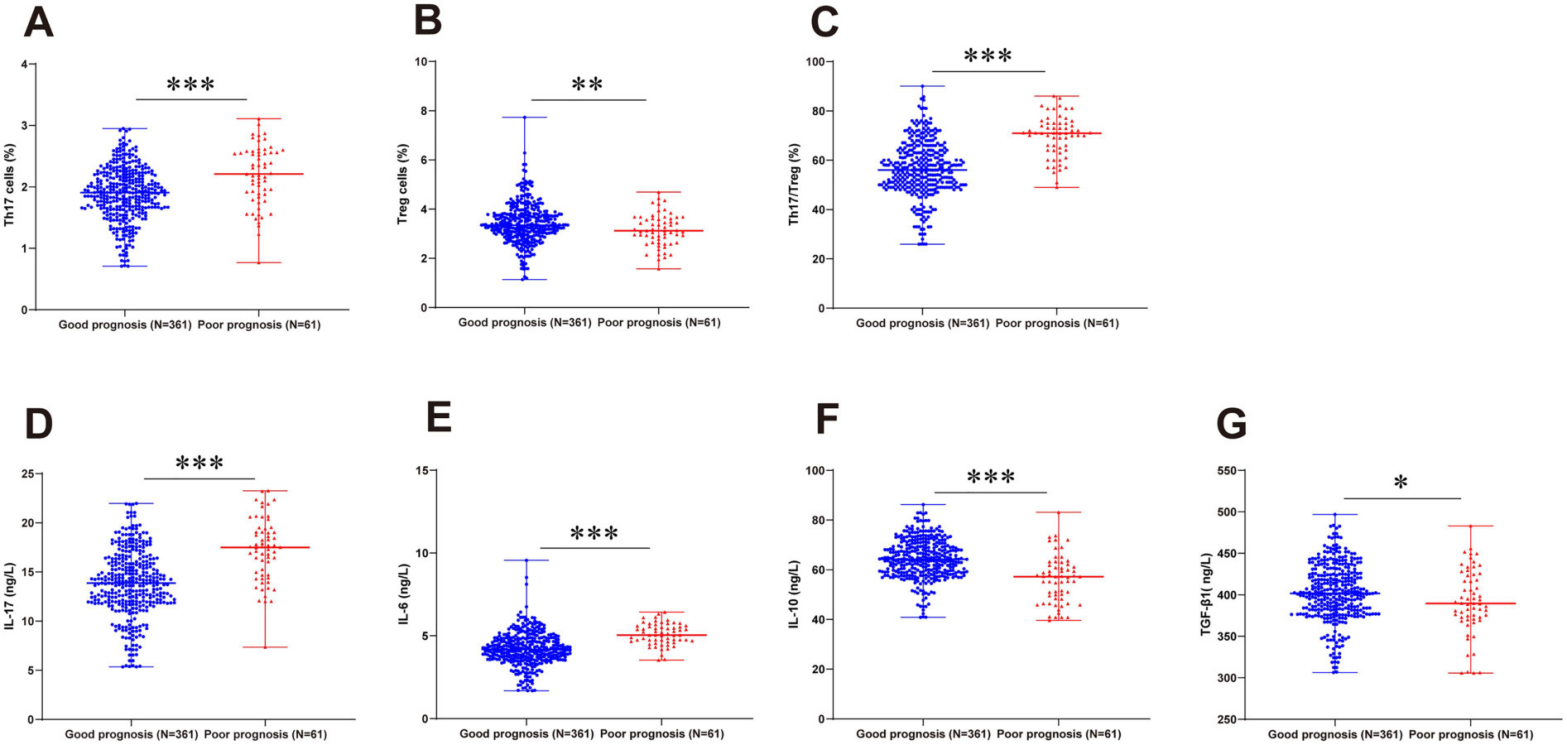
Th17/Treg balance and cytokine profiles stratified by prognostic outcomes. RSVP children were categorized into favorable and unfavorable prognosis groups as per treatment efficacy. Flow cytometric analysis of Th17 **(A)**, Treg **(B)** cell proportions, and calculation of the Th17/Treg ratio **(C)** Serum levels of IL-17 **(D)**, IL-6 **(E)**, IL-10 **(F)**, and TGF-β1 **(G)** determined by ELISA. For panels **(A–G)**, non-normally distributed measurement data were presented as median (minimum, maximum) and compared by the Mann–Whitney *U*-test. * *P* < 0.05, ** *P* < 0.01, *** *P* < 0.001.

Analysis of Th17/Treg balance changes in RSV-infected children with different viral subtypes revealed no statistically significant differences in peripheral blood Th17 and Treg cell levels, Th17/Treg ratios, or serum cytokine expression levels (IL-17, IL-6, IL-10, TGF-β1) among different viral subtypes (all *P* > 0.05; Supplementary Figure S1). Given established evidence linking Th17/Treg imbalance to the occurrence and development of immune-mediated disorders, metabolic diseases, and malignancies (15–19), we further investigated its dynamics relative to disease severity and prognosis in RSVP. The results revealed significantly increased proportions of peripheral blood Th17 cells, elevated serum levels of Th17-associated cytokines (IL-17 and IL-6), and increased Th17/Treg ratios in the moderate-to-severe group vs. the mild group. Conversely, Treg cell proportions and serum levels of Treg-associated anti-inflammatory cytokines (IL-10 and TGF-β1) were substantially lower in moderate-to-severe cases (all *P* < 0.01, Figures 2A–G). Moreover, children with unfavorable prognosis exhibited markedly elevated Th17 cell frequencies, heightened IL-17 and IL-6 levels, and augmented Th17/Treg ratios compared to the favorable prognosis group, while demonstrating suppressed Treg cell proportions and diminished serum IL-10 and TGF-β1 levels (all *P* < 0.05, Figures 3A–G).

### 3.4 Elevated Th17/Treg ratio as an independent risk factor for RSVP

To identify risk factors for unfavorable prognosis in RSVP children, collinearity diagnostics revealed significant multicollinearity among Th17 cells, Treg cells, and the Th17/Treg ratio. Consequently, only the composite Th17/Treg ratio was included in subsequent analyses. Using prognostic outcomes (0 = favorable, 1 = unfavorable) as the dependent variable, univariate logistic regression identified several potential predictors (Table 2). Variables with *P* < 0.05 in the univariate analysis were then entered into multivariate logistic regression as dependent variables. The final model demonstrated that the Th17/Treg ratio (*OR*: 1.135, 95% CI: 1.084–1.188) and serum IL-17 levels (*OR*: 1.411, 95% CI: 1.244–1.601) were independent risk factors for poor prognosis among RSVP children, whereas serum IL-10 levels (*OR*: 0.875, 95% CI: 0.834–0.919) served as an independent protective factor (all *P* < 0.05, Table 2).

**Table 2:** Logistic regression analysis of influencing factors for poor prognosis in RSVP children.

Item	Univariate analysis			Multivariate analysis		
	*P*	*OR*	95% CI	*P*	*OR*	95% CI
Sex (Female = 0, Male = 1)	0.611	0.869	0.504–1.496	–	–	–
Age (months)	0.032	0.871	0.768–0.988	0.742	0.972	0.822–1.150
Current weight (kg)	0.162	0.910	0.798–1.038	–	–	–
Mode of delivery (Vaginal delivery = 0, Caesarean section = 1)	0.375	0.773	0.438–1.365	–	–	–
Gestational age (weeks)	0.100	0.808	0.626–1.042	–	–	–
Birth weight (kg)	0.228	0.564	0.223–1.430	–	–	–
Virus subtype (A = 0, B = 1)	0.349	1.296	0.753–2.232	–	–	–
Fever severity (Moderate = 0, High = 1)	0.069	1.658	0.961–2.860	–	–	–
Respiratory failure (No = 0, Yes = 1)	0.014	3.054	1.256–7.428	0.736	0.815	0.247–2.682
Duration of treatment (≤7 d = 0, >7 d = 1)	<0.001	3.810	2.172–6.682	0.076	1.962	0.932–4.131
IL-17 (ng/L)	<0.001	1.377	1.249–1.518	<0.001	1.411	1.244–1.601
IL-6 (ng/L)	<0.001	2.523	1.818–3.501	0.147	1.344	0.901–2.004
IL-10 (ng/L)	<0.001	0.889	0.857–0.921	<0.001	0.875	0.834–0.919
TGF-β1 (ng/L)	0.026	0.991	0.984–0.999	0.838	0.999	0.990–1.008
Th17/Treg (%)	<0.001	1.132	1.095–1.170	<0.001	1.135	1.084–1.188

### 3.5 Predictive value of Th17/Treg ratio, IL-17, and IL-10 for unfavorable prognosis in RSVP

**Figure 4:**
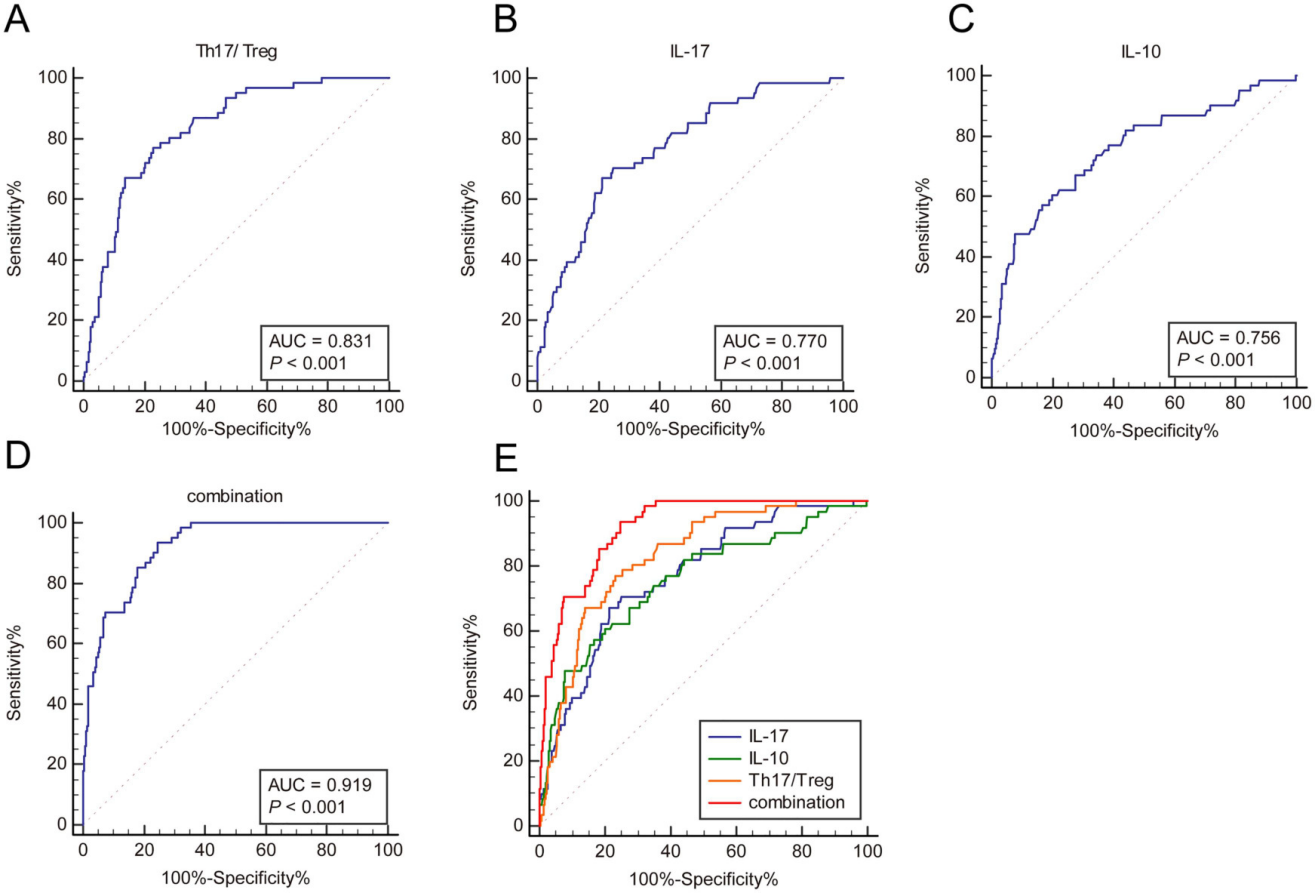
ROC curve analysis of Th17/treg ratio, IL-17, and IL-10 assisting in predict unfavorable prognosis among RSVP children. ROC curves were plotted to evaluate the predictive performance of Th17/Treg ratio **(A)**, IL-17 **(B)**, and IL-10 **(C)** individually, and **(D–E)** their combination for unfavorable prognosis in RSVP children.

Building upon the above findings, the ROC curve analysis was performed to evaluate the auxiliary predictive value of the Th17/Treg ratio, serum IL-17, and serum IL-10, both individually and in combination, for unfavorable prognosis in RSVP children. The Th17/Treg ratio demonstrated an AUC of 0.831 (95% CI: 0.791–0.865) at an optimal cutoff of 64.03%, with 77.05% sensitivity and 77.01% specificity. Serum IL-17 yielded an AUC of 0.770 (95% CI: 0.727–0.810) at a cutoff of 16.41 ng/L (sensitivity: 67.21%; specificity: 78.67%). Serum IL-10 showed an AUC of 0.756 (95% CI: 0.713–0.797) at a cutoff of 57.55 ng/L (sensitivity: 57.38%; specificity: 83.38%). The combination of all three biomarkers achieved a significantly superior AUC of 0.919 (95% CI: 0.889–0.943) with 93.44% sensitivity and 75.35% specificity (all *P* < 0.001, Figure 4). Pairwise comparisons of AUCs using MedCalc confirmed that the combined model significantly outperformed each individual biomarker in predicting unfavorable prognosis in RSVP children (all *P* < 0.001) (Table 3, Figure 4).

**Table 3:** Comparison in predictive performance of biomarkers for unfavorable prognosis among RSVP children.

Indicators	AUC	95% CI	Sensitivity (%)	Specificity (%)
Th17/Treg	0.831	0.791–0.865	77.05	77.01
IL-17	0.770	0.727–0.810	67.21	78.67
IL-10	0.756	0.713–0.797	57.38	83.38
Combination	0.919	0.889–0.943	93.44	75.35
Th17/Treg-Combination	*P* < 0.001			
IL-17-Combination	*P* < 0.001			
IL-10-Combination	*P* < 0.001			

Comparison of AUCs was performed using the DeLong's test.

## 4 Discussion

RSV represents the leading pathogen responsible for ALRTIs in infants and young children. Pneumonia caused by RSV is a major clinical concern in pediatrics due to its rapid progression and high rate of severe cases (20). Although supportive care can improve outcomes for some patients, the lack of effective biomarkers for disease assessment and prognosis prediction remains a significant challenge in clinical management. This study systematically investigated the clinical significance of Th17/Treg immune imbalance in children with RSVP, confirming its close association with disease severity and prognosis, thereby providing new insights into the immunoregulatory mechanisms and clinical management of RSVP.

Th17 and Treg cells, crucial subsets of CD4^+^ T cells, regulate immune responses through a dynamic “pro-inflammatory and anti-inflammatory” balance (21). Th17 cells primarily secrete pro-inflammatory cytokines like IL-17 and IL-6, facilitating the clearance of extracellular pathogens and mediating inflammatory responses. Conversely, Treg cells maintain immune homeostasis by suppressing excessive immune activation through the secretion of anti-inflammatory cytokines such as IL-10 and TGF-β1 (22). Our findings revealed a significant increase in the proportion of peripheral blood Th17 cells and the Th17/Treg ratio in RSVP children, accompanied by elevated levels of IL-17 and IL-6. Concurrently, the proportion of Treg cells and the levels of IL-10 and TGF-β1 were significantly reduced. It is indicated that RSV infection disrupts this equilibrium, inducing a state of immune dysregulation characterized by exaggerated pro-inflammation and insufficient anti-inflammation. The mechanism underlying this phenomenon may involve RSV stimulation of airway epithelial cells. Following infection, these cells release chemokines like IL-1β and IL-10, which promote the differentiation of naive CD4^+^ T cells towards the Th17 lineage while suppressing the differentiation and function of Treg cells (5). Overactivated Th17 cells recruit neutrophils into lung tissue via IL-17, and these neutrophils release oxygen free radicals and matrix metalloproteinases, exacerbating alveolar epithelial damage and airway constriction (23). Concurrently, defective Treg cell function weakens the suppression of the inflammatory cascade, creating a vicious cycle (24). Previous research has demonstrated that RSV infection significantly reduces the proportions of Foxp3^+^ Treg cells, increases the proportions of Th17 cells, and inhibits the conversion of Th17 cells into Foxp3^+^ Treg cells (25). An animal study confirms increased Th17 cell infiltration and decreased Treg cells in the lungs of RSV-infected mice, correlating with the severity of alveolitis (11). Similarly, clinical data indicate that the Th17/Treg ratio and IL-6 levels were higher in the RSVP group compared to groups with common pneumonia and healthy controls (10). Our study validated these findings in a clinical pediatric cohort, providing direct evidence for the immunopathogenesis of RSVP. This finding resonates with recent observations by Gambadauro et al., which confirmed that the severity of bronchiolitis—primarily caused by RSV—significantly impacts peripheral blood cell counts and levels of conventional inflammatory markers such as CRP (26). However, our study extends beyond this. While Gambadauro et al. revealed the macro-level association between disease severity and systemic inflammatory status, our findings pinpoint the underlying immunopathological core driving this inflammatory state and tissue damage—namely, the imbalance between Th17 (pro-inflammatory) and Treg (anti-inflammatory) cells. This indicates that behind the abnormalities in conventional inflammatory markers lies a more intricate disorder of adaptive immune regulation. This association suggests that developing a comprehensive prognostic assessment model holds significant potential for the future. This model could integrate readily available conventional parameters (e.g., CRP, neutrophil count) for rapid screening, supplemented by more pathologically specific immunological markers (e.g., Th17/Treg ratio) for precise risk stratification. This dual-track strategy of conventional + specific holds promise for earlier and more accurate identification of high-risk pediatric patients without unduly increasing the burden on healthcare systems.

Critically, we found that the degree of Th17/Treg imbalance was significantly correlated with disease severity in RSVP children. Patients in the moderate-to-severe group exhibited significantly higher proportions of Th17 cells, Th17/Treg ratios, and levels of IL-17 and IL-6, alongside significantly lower proportions of Treg cells and levels of IL-10 and TGF-β1 than in the mild group. This suggested that the Th17/Treg ratio could serve as a quantitative indicator for assessing disease severity. Pathophysiologically, excessively elevated IL-17 promotes airway smooth muscle cell proliferation and mucus hypersecretion by activating the nuclear factor kappa B pathway, thereby worsening airway obstruction (27). Reduced IL-10 levels impair the effective suppression of pro-inflammatory cytokine release, leading to uncontrolled inflammation, which is closely associated with complications like respiratory failure and heart failure frequently observed in moderate-to-severe cases (28). More importantly, children with poor prognosis displayed significantly elevated Th17/Treg ratios and IL-17 levels, coupled with significantly decreased IL-10 levels. Multivariate logistic regression analyses confirmed that an elevated Th17/Treg ratio and increased IL-17 levels were independent risk factors for poor prognosis, while elevated IL-10 levels served as an independent protective factor. These results demonstrate that Th17/Treg dysregulation not only participates in the pathological process of RSVP but also acts as a crucial prognostic indicator. For instance, IL-17, the signature cytokine of Th17 cells, indicates irreversible lung tissue damage with its sustained high expression (29). Conversely, low levels of IL-10, the core anti-inflammatory cytokine of Treg cells, reflect impaired immune regulatory capacity, making it difficult to control the post-infection cytokine storm (30). Effective prognostic prediction tools are essential for risk stratification and individualized treatment of RSVP children. Our ROC curve analysis demonstrated that the Th17/Treg ratio predicted poor prognosis with an AUC of 0.831, outperforming IL-17 (AUC = 0.770) and IL-10 (AUC = 0.756) individually. Remarkably, the combined detection of all three markers achieved an AUC of 0.919, significantly enhancing predictive power. This advantage may stem from the complementary nature of these indicators. The Th17/Treg ratio reflects the overall state of immune dysregulation, IL-17 quantifies pro-inflammatory intensity, and IL-10 assesses anti-inflammatory capacity; together, they provide a more comprehensive picture of the immune functional status in RSVP children. Compared to traditional inflammatory markers such as CRP and PCT, Th17/Treg-related indicators more specifically reflect dysregulation in immune modulation, whereas traditional markers only indicate the general level of inflammation (31). The combined model (Th17/Treg ratio + IL-17 + IL-10) achieved high sensitivity of 93.44% in our study, enabling effective identification of the vast majority of high-risk children. The findings of this study provide new insights into the clinical management of RSVP, primarily reflected in the following aspects, which may serve as a reference for clinicians and facilitate translation into clinical practice. (1) Early risk stratification and precision monitoring: Currently, clinicians primarily rely on clinical symptoms and conventional inflammatory markers (such as CRP) to assess RSVP disease severity. Our study introduces a novel, more immunopathologically specific biomarker panel (Th17/Treg, IL-17, IL-10). Clinicians can utilize this panel, particularly during early hospitalization, to perform more precise risk stratification of pediatric patients. This enables identification of high-risk children, optimization of resource allocation (e.g., more frequent vital sign monitoring, earlier assessment of respiratory support needs), and prevention of rapid deterioration. (2) Family communication: This biomarker combination (particularly the high AUC value of the model compromising Th17/Treg, IL-17, IL-10) provides a more objective prediction of the likelihood of RSV-infected children developing adverse outcomes. This assists physicians in setting reasonable treatment expectations and enables them to explain the severity of the condition and potential disease course to families in a more scientifically grounded manner. (3) Targets and rationale for future immunomodulatory therapies: This represents the most pivotal clinical insight. Our findings go beyond serving as a mere predictive tool, revealing that the Th17/Treg axis constitutes a potential therapeutic target for RSVP. Although no targeted drugs are currently in routine clinical use, this discovery charts a course for future precision medicine. Physicians and researchers can explore whether existing immunomodulators can be used as adjunctive therapy for high-risk pediatric patients (those with significant Th17/Treg imbalance) to restore immune balance and improve prognosis. This lays the theoretical foundation for developing novel RSVP therapies, driving a paradigm shift in treatment from symptomatic support to immune regulation.

The novelty of this study lies in being the first to systematically demonstrate the independent association between peripheral blood Th17/Treg immune imbalance and both disease severity and prognosis in RSVP children. Furthermore, it establishes the superior prognostic predictive value of combining the Th17/Treg ratio, IL-17, and IL-10, offering a novel panel of immune biomarkers for clinical use. However, limitations still exist. First, this single-center retrospective study inherently limits the ability to establish causal inferences between Th17/Treg imbalance and RSV disease severity or prognosis. Additionally, this design may introduce selection bias that cannot be entirely avoided. Second, the study's focus was relatively narrow, selecting only the most representative Th17 and Treg-related parameters for investigation, without simultaneously evaluating broader systemic inflammatory markers (such as IL-1β, TNF-α, etc.) and conventional hematological indicators (such as the neutrophil-lymphocyte ratio). This limitation partially restricts the ability to comprehensively interpret Th17/Treg immune imbalance within the broader context of the systemic immune response to RSV infection. Third, testing was conducted using a single blood sample collected prior to treatment, lacking longitudinal follow-up data on the dynamic changes in Th17/Treg balance during treatment and recovery. Consequently, it was impossible to reveal the temporal relationship between immune recovery and clinical improvement. Furthermore, the mechanism underlying the role of Th17/Treg immune balance in RSVP pathology remains incompletely understood, necessitating broader and more in-depth research. Finally, and critically, the combined predictive model based on the Th17/Treg ratio, IL-17, and IL-10 demonstrated good discriminatory performance in this cohort. However, it has not yet undergone external validation in an independent, multicenter cohort. This limitation restricts the model's current general applicability, and its robustness and efficacy prior to clinical implementation require further confirmation in prospective studies. Future studies should expand sample sizes, conduct multicenter research and external validation to enhance test sensitivity and result reliability. Future investigations should incorporate broader indicators (such as neutrophil-to-lymphocyte ratio, other cytokine profiles, routine hematological parameters, etc.) to interpret Th17/Treg imbalance within a broader immunological context and further explore the mechanisms by which Th17/Treg immune balance contributes to the onset and progression of RSV infection.

## Data Availability

The original contributions presented in the study are included in the article/Supplementary Material, further inquiries can be directed to the corresponding author.

## Supplementary material

Supplementary Figure S1 Comparison of Th17/Treg cells and cytokines in RSV-infected children with different viral subtypes. Children with RSV infection were divided into Group A and Group B based on viral subtype. Flow cytometry was used to detect the proportion of peripheral blood Th17 **(A)** and Treg **(B)** cells, and the Th17/Treg ratio **(C)** was calculated. ELISA was used to detect serum IL-17 **(D)**, IL-6 (E), IL-10 **(F)**, and TGF-β1 **(G)** levels. For non-normally distributed quantitative data **(A–G)**, median values (min, max) were used. Comparisons between groups employed the Mann-Whitney *U*-test; ns indicates *P* > 0.05.
