# Supplementary figures and images for: Predictive value of Th17/Treg immune imbalance for disease severity and poor prognosis in children with respiratory syncytial virus pneumonia
Source: Front Pediatr. 2025 Dec 4;13:1725402. doi: 10.3389/fped.2025.1725402 (PMC12711749; doi:10.3389/fped.2025.1725402)

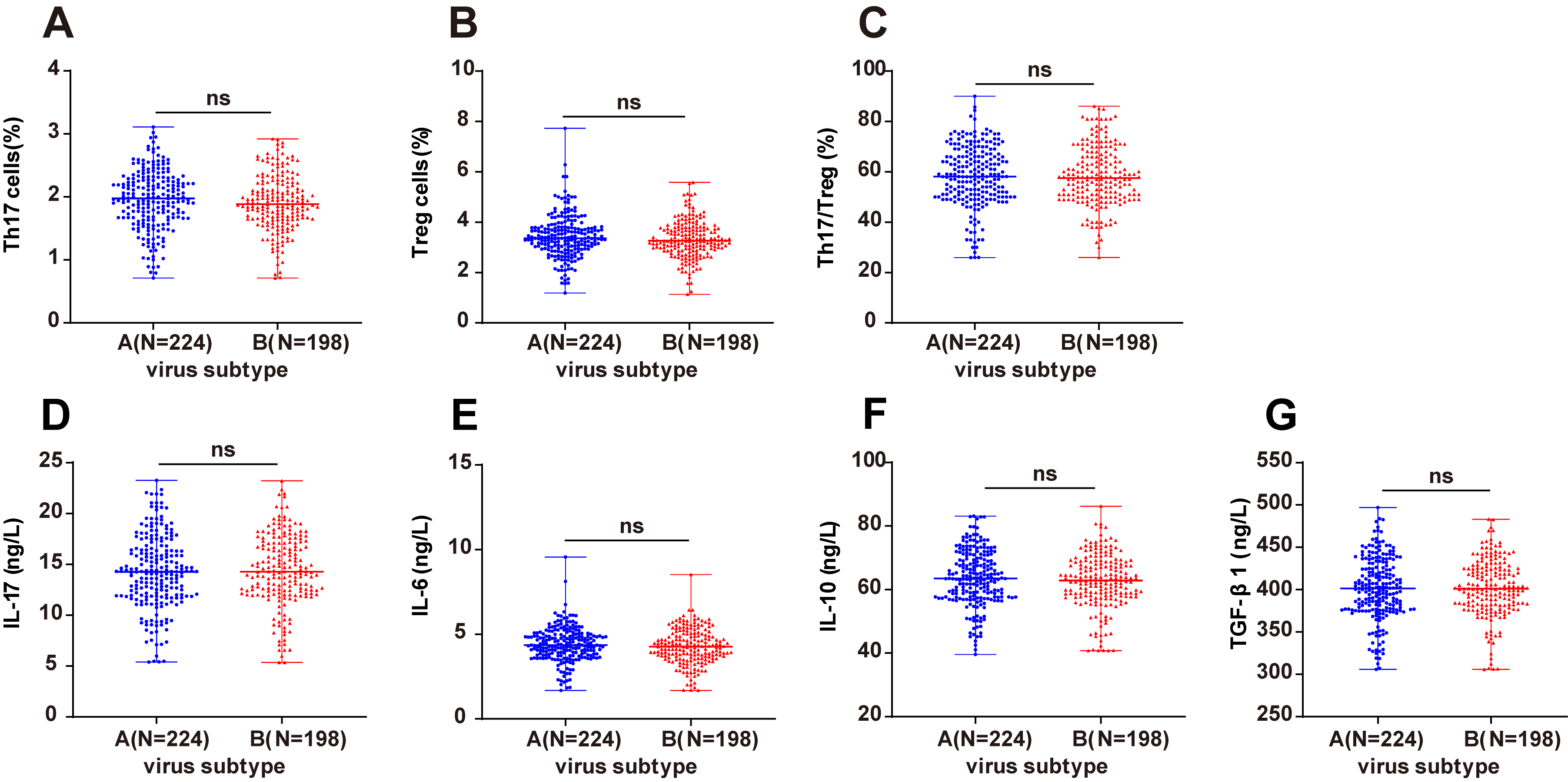

Supplement: Supplementary Figure S1 — Comparison of Th17/Treg cells and cytokines in RSV-infected children with different viral subtypes. Children with RSV infection were divided into Group A and Group B based on viral subtype. Flow cytometry was used to detect the proportion of peripheral blood Th17 (A) and Treg (B) cells, and the Th17/Treg ratio (C) was calculated. ELISA was used to detect serum IL-17 (D), IL-6 (E), IL-10 (F), and TGF-β1 (G) levels. For non-normally distributed quantitative data (A–G), median values (min, max) were used. Comparisons between groups employed the Mann-Whitney U-test; ns indicates P > 0.05. [file Image1.tif]
